# Evidence-Based Recommendations on the Use of Immunotherapies and Monoclonal Antibodies in the Treatment of Male Reproductive Cancers

**DOI:** 10.3390/curroncol32020108

**Published:** 2025-02-14

**Authors:** Farhan Khalid, Zubair Hassan Bodla, Sai Rakshith Gaddameedi, Raymart Macasaet, Karan Yagnik, Zahra Niaz, Peter N. Fish, Doantrang Du, Shazia Shah

**Affiliations:** 1 Department of Internal Medicine, Monmouth Medical Center, Long Branch, NJ 07740, USAsai.gaddameedi@rwjbh.org (S.R.G.); karanj.yagnik@rwjbh.org (K.Y.);; 2 Department of Internal medicine, Graduate Medical Education, College of Medicine, University of Central Florida, Orlando, FL 32827, USA; 3 Department of Medicine, Al-Aleem Medical College, Lahore 54000, Pakistan

**Keywords:** genitourinary carcinomas, monoclonal antibodies, treatment modalities, immunotherapy, prostate cancer, male reproductive tract cancers

## Abstract

The incidence of male reproductive cancers, including prostate, testicular, and penile cancers, has risen in recent years, raising important health concerns. Prostate cancer is the second leading cause of cancer-related mortality in men, while penile cancer, though rare, typically affects men over 60. Testicular cancer, with a lifetime risk of about 0.4% in men, is most common among adolescents and young adults, decreasing sharply after the age of 40. Traditional treatments include chemotherapy, radiation, surgery, and combinations thereof, but advancements in immunotherapy and monoclonal antibodies are showing promising results, particularly for genitourinary cancers. These therapies, targeting immune checkpoints and tumor-specific antigens, are gaining traction as effective alternatives for resistant cases. This review provides evidence-based recommendations on current and emerging immunotherapy and monoclonal antibody treatments for male reproductive cancers, highlighting future directions to optimize patient outcomes.

## 1. Introduction

Genital cancers or reproductive tract cancers in male encompass tumors of the prostate, testes, and penis. Overall, prostate cancer is the second most common cancer in males after skin cancers, and, furthermore, the incidence rate of prostate cancer has increased by 3% per year since 2014, as per the latest American Cancer Society (ACS, 2023) statistics. On the other hand, penile cancer is one of the rarer cancers in the USA, with the incidence being as low as less than 1 in 100,000 population as per the cancer facts and figures report, 2023, by the American Cancer Society. Nearly all testicular tumors are germ-cell tumors, and are more common in adolescent and young males, but incidence declines rapidly after the age of 40 [1]. Traditional approaches like surgery, chemotherapy, and radiation have certain limitations, forcing us to explore new and innovative treatments such as immunotherapy. Immunotherapy, which leverages the body’s immune system and targets and combats cancer cells, has emerged as a promising avenue for improved outcomes and reduced side-effects in the realm of male genital cancers.

Immune checkpoint and cancer-testis antigens (CTAs) are sensible targets for immune therapy [2,3]. The current checkpoint inhibitors target PD-1 and CTLA-4, two immune checkpoints, with good clinical efficacy. Activated T-cells express PD-1 and its ligand, PD-L1, which modulates T-call activity to inhibit the immune response. When expressed by activated T cells, CTLA-4, an immunoglobulin, downregulates immune response by binding to B7. These therapies had a 20% objective response rate and a 1-year response durability [4,5,6]. Recent developments in anti-cancer therapy have seen the employment of CTA-focused antibodies, vaccines, and chimeric antigen receptor-modified T cells (CAR-T), which have shown encouraging results in preclinical studies and initial clinical trials [3].

Monoclonal antibodies target these immune checkpoints, PD-1, PDL-1, and CTLA-4, and target cancer cells by upregulating T-cells [7,8,9]. Moreover, monoclonal antibodies (mAB) therapy continues to evolve, and new discoveries in these areas of cancer therapy show promise for the future.

### 1.1. Prostate Cancer

#### 1.1.1. Introduction and Overview of Prostate Cancer

The second leading cause of death from cancer in men is prostate cancer, which is the most prevalent noncutaneous cancer in men in the United States [10].

#### 1.1.2. Epidemiology of Prostate Cancer

The prostate cancer incidence was 268,490 in 2022, and the 5-year relative survival was 96.8%. However, the 5-year relative survival for those with distant metastases fell precipitously to 32.3% [11]. Although prostate cancer treatment methods have advanced greatly in recent years, patients with metastatic castration-resistant prostate cancer (mCRPC) still have a dismal prognosis [12].

#### 1.1.3. Etiology and Risk Factors

In contrast to other frequent tumors, prostate cancer etiology is still a mystery. Some established risk factors for prostate cancer include advanced age, a positive family history, prostate inflammation, obesity, a lack of activity, ethnicity, and persistently high testosterone levels.

### 1.2. Treatment Modalities for Prostate Cancer

#### 1.2.1. Localized Prostate Cancer

Active surveillance, radical prostatectomy, external radiation, and brachytherapy are the main treatments used to manage localized prostate cancer. However, there is evidence of biochemical malignancy recurrence seen after ten years of initial treatment in roughly 30–50% of patients who received radiation or in 20–40% of patients who underwent prostatectomy [13,14].

#### 1.2.2. Androgen Deprivation Therapy (ADT)

Since prostate cancer is an androgen-dependent cancer, the majority of first-line treatments concentrate on suppressing androgen synthesis and the androgen receptor (AR) signaling axis [15]. Androgen deprivation therapy (ADT) paired with an additional therapy, such as an AR-signaling inhibitor (ARSI) or chemotherapy, is the first-line treatment for metastatic prostate cancer [16]. Castration-resistant prostate cancer is a disease condition marked by eventual resistance, despite the fact that ADT is extremely effective in the majority of patients (Castration-resistant prostate cancer (CRPC)).

#### 1.2.3. Advances in Systemic Therapies for CRPC

Patients with CRPC now have better overall survival (OS) thanks to the expansion of systemic medicines that target PC outside of the AR-signaling axis throughout the past ten years of study [10,17,18,19].

### 1.3. Immunotherapy in Prostate Cancer

#### 1.3.1. Introduction to Immunotherapy

Immunotherapy, a therapeutic approach with several Food and Drug Administration (FDA) approvals in recent years, shows promise for a range of tumor types.

#### 1.3.2. Immune Checkpoint Inhibitors (ICIs)

Some of the immunotherapies like immune checkpoint inhibitors (ICIs) target the proteins programmed death-1 (PD-1)/programmed death-ligand 1 (PD-L1) and cytotoxic T lymphocyte-associated antigen 4 (CTLA-4), and have completely changed the way that genitourinary malignancies like renal cell carcinoma and urothelial carcinoma are treated [12]. Immunotherapy played a major role in these advancements of treatment in prostate cancer. Some of the newer immunotherapy treatments for prostate cancers are Sipuleucel T immunotherapy and immune checkpoint inhibitors like Pembrolizumab, Nivolumab, ipulimumab, atezolizumab, CAR-T cell therapy, and BITE-T Cell therapy. Below, we discuss the recent advancements in the field of immunotherapy in regard to prostate cancer management and their clinical trials.

#### 1.3.3. Specific Immunotherapies for Prostate Cancer

##### Sipuleucel-T Immunotherapy

Sipuleucel-T (sip-T), an anti-prostatic acid phosphatase therapeutic autologous cellular vaccination, is the only immunotherapy FDA-approved to date for metastatic castration-resistant prostate cancer (mCRPC) (Prostatic acid phosphatase (PAP)). Sip-T is formed through the ex vivo cultivation of patients’ peripheral blood mononuclear cells (PBMC) using a fusion protein of human recombinant PAP and granulocyte-macrophage colony-stimulating factor (GM-CSF), which is known as PA2024, which leads to the activation of cells that present antigens. Then, over the course of three doses, the patients receive the activated cellular product again [20].

### 1.4. Clinical Trials and Evidence for Sipuleucel-T

#### 1.4.1. Overview of Sipuleucel-T Trials

In one study with 127 participants having metastatic castration-resistant prostate cancer, conducted as a randomized, placebo-controlled trial, there was a 41% decrease in mortality risk among the patients treated with sipuleucel-T compared to the placebo group (with a hazard ratio of 0.59 for the sipuleucel-T group; 95% confidence interval [CI] ranging from 0.39 to 0.88; *p* = 0.01). In another study, also randomized and placebo-controlled, indicated a possible improvement in survival rates for those patients who are treated with sipuleucel-T, though the results did not reach statistical significance. None of these studies demonstrated a marked impact on delaying the progression of the disease, which was the main goal of both trials [10,21].

#### 1.4.2. Phase III Trials

Phase III trials are pivotal studies designed to evaluate the safety and efficacy of a treatment in a larger patient population. The following section highlights key Phase III trials conducted to assess the effectiveness of sipuleucel-T in metastatic prostate cancer.

##### D9901 Trial

In the D9901 trial, 127 patients with metastatic, asymptomatic prostate cancer were randomized in a 2:1 ratio to receive either sipuleucel-T (82 patients) or a placebo (45 patients) in three bi-weekly infusions. Patients on placebo could later receive APC8015F if their disease progressed. Over a 36-month follow-up, median time to disease progression (TTP) was slightly longer with sipuleucel-T (11.7 weeks vs. 10.0 weeks; *p* = 0.052), but this result was not statistically significant. However, sipuleucel-T significantly improved median survival (25.9 vs. 21.4 months; *p* = 0.01). T-cell stimulation in sipuleucel-T recipients increased eightfold by week 8, suggesting a potential survival benefit for asymptomatic prostate cancer patients [21].

##### D9902A Trial

Enrollment in D9902A was suspended after 98 patients were randomly assigned to receive sipuleucel-T (*n* = 65) or a placebo (*n* = 33) at 27 clinical research locations across the United States between May 2000 and March 2003. The time to disease progression was not statistically different across the therapy groups, despite the HR favoring the sipuleucel-T arm (HR, 1.09; 95% confidence interval [CI], 0.69–1.70; *p* = 0.72). Compared to placebo, sipuleucel-T reduced the risk of death by 21%; however, this difference was not statistically significant (HR, 1.27; 95% CI, 0.78–2.07; *p* = 0.33). Less well-balanced baseline prognostic variables existed in D9902A compared to D9901. The survival treatment effect in D9902A was comparable to that seen in D9901 after adjusting for baseline prognostic variables [22,23].

##### Combined Analysis of D9901 and D9902A

The combined analysis of the D9901 and D9902A phase 3 trials, both randomized, double-blind, and placebo-controlled, evaluated sipuleucel-T in advanced prostate cancer patients. Out of 225 participants, 147 received sipuleucel-T in three bi-weekly infusions, while 78 received a placebo. Over a follow-up period of up to 36 months, sipuleucel-T treatment reduced the risk of death by 33% (hazard ratio, 1.50; 95% CI, 1.10–2.05; *p* = 0.011). Additionally, there was a non-significant 21% reduction in disease progression. Although sipuleucel-T extended survival, it did not significantly impact the time to disease progression, prompting the subsequent IMPACT trial to further assess overall survival benefits [23].

##### IMPACT Trial (D9902B)

In a phase 3, double-blind, placebo-controlled trial of 512 men with metastatic castration-resistant prostate cancer, the participants were randomly assigned to receive either sipuleucel-T or a placebo in a 2:1 ratio. Sipuleucel-T was administered in three intravenous infusions at two-week intervals, aiming to improve overall survival. The results indicated a 22% reduction in the risk of death for the sipuleucel-T group, with a median survival increase of 4.1 months (25.8 vs. 21.7 months for placebo), and a higher 36-month survival probability (31.7% vs. 23.0%). Although adverse events such as chills, fever, and headache were more common in the sipuleucel-T group, the therapy successfully extended overall survival without altering disease progression rates. Supporting phase 1 and 2 studies also showed decreased PSA levels, confirming sipuleucel-T’s immunologic and therapeutic benefits.

#### 1.4.3. Phase I and II Studies

The study was designed to evaluate safety, dosage, and immune response. Transient symptoms such as fever, chills, muscle pain, discomfort, tiredness, urgent need for urination, urinary incontinence, and frequent nighttime urination were observed. They were able to reveal a decrease in PSA levels after sipuleucel-T. T cells drawn from patients after infusion with sipuleucel-T could be stimulated by GM-CSF in vitro, unlike placebo [23].

### 1.5. Immune Checkpoint Inhibitors in Prostate Cancer

#### 1.5.1. Pembrolizumab

Pembrolizumab, the only FDA-approved immune checkpoint inhibitor (ICI) for prostate cancer, targets PD-1 but is specifically approved for metastatic castration-resistant prostate cancer (mCRPC) patients with high microsatellite instability (MSI-H), deficient mismatch repair (dMMR), or a tumor mutational burden (TMB) of at least 10 mutations per megabase, particularly as a subsequent treatment after docetaxel and hormonal therapy failure. The phase II KEYNOTE-158 study found a clinical benefit in patients with MSI-H and dMMR tumors, demonstrating significant responses in those with TMB of 10 mut/Mb (20 of 102 patients and 43 of 688 patients, respectively) [24,25].

Another study with anti-PD1 therapy in 65 patients with dMMR mCRPC showed a median progression-free survival (PFS) of 24 weeks, with a 65% PSA50 response rate [26].

In an additional trial, pembrolizumab was administered to 258 mCRPC patients, with results indicating modest responses in PSA and objective response rates for those on monotherapy; however, the cohort receiving both enzalutamide and pembrolizumab had a higher overall response rate (ORR) compared to monotherapy alone, suggesting enhanced effectiveness in combination therapy [27].

#### 1.5.2. Ipilimumab

In two sizable phase III trials, both chemotherapy-naive and docetaxel-pretreated mCRPC patients received ipilimumab monotherapy. Ipilimumab prolonged PFS and PSA in the group of patients with mCRPC, even though there was no discernible difference between the ipilimumab group and the placebo group in terms of overall survival in the primary analysis [28,29].

#### 1.5.3. Atezolizumab and Nivolumab

Atezolizumab demonstrated a minimal PSA response rate of 8.6% in a phase 1 study that looked at its use in 35 mCRPC patients who had progressed on sipuleucel-T or enzalutamide [30]. Combination with enzalutamide was studied in the IMbassador250 trial which did not meet the primary end points.

Patients with melanoma, non-small-cell lung cancer, mCRPC, renal cell carcinoma, and colorectal carcinoma were enrolled in a phase 1 trial that examined nivolumab. However, nivolumab monotherapy did not produce any objective responses in the patient population with prostate cancer [9].

#### 1.5.4. Combination Therapies

The phase III IMbassador250 study compared atezolizumab plus enzalutamide to enzalutamide alone in 759 patients with mCRPC who had progressed on abiraterone and were ineligible for taxane-based therapy. The study did not achieve its primary endpoint of improved overall survival for the combination therapy group [31]. Similarly, the phase III KEYNOTE-641 trial, which evaluated pembrolizumab combined with enzalutamide and androgen deprivation therapy (ADT) in mCRPC patients, was halted early due to lack of improvement in radiographic progression-free survival (rPFS) and overall survival (OS) [32]. In the phase II CheckMate 9KD trial, nivolumab and docetaxel were administered to 41 mCRPC patients receiving continuous ADT, resulting in a PSA response of 46.3% and an overall response rate (ORR) of 36.8% in patients with detectable illness. This has led to further exploration of immunotherapy’s role alongside docetaxel in the ongoing phase III CheckMate 7DX trial [33].

Additional combination therapies have been tested, such as pembrolizumab with docetaxel and prednisone in chemotherapy-naive mCRPC patients, showing a 28% PSA response rate and an 18% ORR in cohort B of the KEYNOTE-365 trial [34]. However, in the phase III KEYNOTE-921 trial of pembrolizumab, docetaxel, and prednisone, the primary rPFS and OS endpoints were not met [35]. Dual immune checkpoint blockade was also investigated in the phase II CheckMate 650 trial, where ipilimumab and nivolumab showed clinical activity in mCRPC patients, especially those with high tumor mutational burden (TMB), though treatment was stopped due to early toxicity [36]. Recent ASCO GU 2023 findings on ipilimumab and nivolumab, as well as ipilimumab with cabazitaxel, showed that some patients achieved a significant reduction in tumor size and PSA (75–100%), underscoring the potential for continued exploration in carefully selected patients [37] (Table 1).

**Table 1:** Combination therapies for prostate cancer management [24].

Serial Number	Study	Management	Result
1	Phase III IMbassador250 trial	Atezolizumab in combination with Enzalutamide versus Enzalutamide treatment alone	Did not achieve the primary endpoint of increased overall survival.
2	Phase III KEYNOTE-641 trial	Pembrolizumab combined with enzalutamide and androgen deprivation therapy (ADT) in metastatic castration-resistant prostate cancer (mCRPC) patients.	After an interim investigation revealed no improvement in rPFS or OS, ceased.
3	Phase II CheckMate 9KD trial	Utility of nivolumab and docetaxel on ADT in chemo-naive mCRPC patients	The PSA response was 46.3 percent, but the ORR in patients with detectable illness was 36.8 percent.
4	KEYNOTE-365 trial (cohort B)	Combination therapy with Pembrolizumab plus Docetaxel and Prednisone in chemotherapy-naive mCRPC patients	Among the 104 patients who received treatment, a PSA response was observed in 28% of the cases, accompanied by an overall response rate of 18%.
5	Phase III KEYNOTE-921 trial	Pembrolizumab and Docetaxel and Prednisone in chemotherapy-naive mCRPC patients	Primary endpoints of rPFS and OS were not met.
6	Phase II CheckMate 650 trial	Significance of ipilimumab and nivolumab in mCRPC patients previously undergoing docetaxel treatment.	Treatment discontinuation occurred due to early toxicity.

### 1.6. Advanced Cellular Therapies in Prostate Cancer

#### 1.6.1. Chimeric Antigen Receptor (CAR) T-Cell Therapy

The therapeutic approach involving chimeric antigen receptor-modified T cells (CAR-T) has shown great success in treating blood cancers and holds great promise for application in future cancer care. These chimeric antigen receptors (CARs) are specifically engineered cell receptors designed to enable lymphocytes (primarily T cells) to detect and destroy cancer cells that display relevant antigens, following the deliberate introduction of specific tumor-associated antigens (TAAs) [38].

#### 1.6.2. PSMA-Targeted CAR T-Cell Therapy

Currently identified tumor-associated antigens (TAAs) for prostate cancer (PCa) include prostate acid phosphatase (PAP), transient receptor potential (Trp)-p8, prostate-specific membrane antigen (PSMA), prostate stem cell antigen (PSCA), and prostate-specific antigen (PSA) [39].

Prostate-specific membrane antigen (PSMA) is one of the significant prostate-specific markers. Two studies, Zuccolotto et al. and Wang et al., have found considerable success in using CART-T cell therapies in mice. Tumor eradication was seen in the mice [40,41].

Treatment of mCRPC and evaluation of CAR-Ts with dominant-negative TGFβ receptors were performed in a phase I clinical study (NCT03089203) of PSMA-targeting TGFβ-insensitive armored CAR-Ts. These results confirmed their safety and feasibility [42]. P-PSMA-101 was used in the highly anticipated open-label, multicenter, 3 + 3 dose-escalating phase I trial, which has been reported. Circulating tumor cells (CTCs) have decreased concurrently with its use, and a post-treatment tumor biopsy showed P-PSMA-101 CAR T-cell infiltration and a confirmed pathologic full response [43].

Recently, several clinical trials of PSMA-targeted CAR-T immunotherapies have been conducted. Some of the PSMA CART cell clinical trials currently on going are as below Table 2.

#### 1.6.3. Prostate Stem Cell Antigen (PSCA) Targeted CAR T-Cell Therapy

One of the most well-known prostate-specific markers is PSCA, a tiny glycoprotein of 123 amino acids that is a member of the Thy-1/Ly-6 family of glycosylphosphatidylinositol (GPI)-anchored cell surface antigens. Because of its prostate-specific characteristics, PSCA is crucial in PCa immunotherapy. It increases with tumor staging, is overexpressed in the majority of prostate cancers, and is substantially expressed in prostate cancer bone metastases [30]. Priceman et al. created two types of PSCA-CAR, CD28-PSCA-CAR and 4-1BB-PSCA-CAR. Both these PSCA-CARs have robust in vivo antitumor activity in patient-derived bone-metastatic prostate cancer xenograft models, but 4-1BB showed better control of disease in comparison to CD28 [44]. Currently, NCT03873805 PSCA-CAR-T I, March 13, 2019, with the estimated completion year of 2023, is an ongoing clinical trial regarding PSCA CART therapy.

#### 1.6.4. Prostate-Specific Antigen (PSA) and Other TAAs

PSA is a kallikrein-like serine protease that is almost exclusively expressed by prostate epithelial cells and secreted into the seminal fluid to trigger particular T-cell reactions [45]. The preferred target for CAR-T treatment is PSA because of its prostate-specificity. But PSA is also increased in malignancies other than prostate cancer. PSA precursor (also known as proPSA) and its derivatives have the potential to be used as new CAR-T targets for prostate cancer because it has been demonstrated that they can identify prostate cancer from non-cancer more effectively [46].

Prostatic Acid Phosphatase (PAP), Epithelial Cell Adhesion Molecule (EpCAM), and Transient Receptor Potential (Trp-p8) are other TAAs that are currently being studied which have the potential to become new CAR-T targets for prostate cancer. Currently, NCT03013712 EpCAM -CAR I/II, 6 January 2017, is an ongoing clinical trial regarding EpCAM CART therapy [38].

### 1.7. Bispecific T-Cell Engager (BiTE) Therapy

#### 1.7.1. Overview and Mechanism of BiTE Therapy

The immunotherapy known as bispecific T-cell engager (BiTE) is an alternate method for T-cell re-routing in solid tumor malignancies. Select hematologic malignancies have benefitted significantly from this treatment class, and BiTE constructs are currently the subject of ongoing research for metastatic prostate cancer.

BiTE immunotherapies are separate antibody constructions that include two different epitopes, one of which has affinity for TAAs and the other for CD3ζ on T cells. An immunological synapse may form on the surface of tumor cells as a result of the BiTE molecule acting as a physiologic link between the tumor cell and the effector T-cell [47].

#### 1.7.2. Key Clinical Trials and Outcomes

Patients with advanced CRPC underwent a further phase I, open-label, dose-escalation study of pasotuxizumab to observe responses to PSA and other modalities. For treatment with subcutaneous and intravenous pasotuxizumab, the research involved 31 and 16 patients, respectively. In the cIV group, the PSA decreased over the course of the study’s treatment in 14 out of 16 individuals. According to exploratory research, cIV therapy also resulted in a dose-dependent decrease in detectable circulating tumor cells [48].

### 1.8. Summary and Future Directions in Immunotherapy for Prostate Cancer

Recent advancements in immunotherapy for prostate cancer, particularly for metastatic castration-resistant prostate cancer (mCRPC), highlight promising treatment options like Sipuleucel-T and checkpoint inhibitors, though response rates are generally modest. Combination therapies and emerging approaches such as CAR T-cell and BiTE therapies show potential but require further study for efficacy and safety. Future research should focus on optimizing combination therapies, identifying responsive patient profiles, and refining CAR T-cell and BiTE protocols to overcome tumor microenvironment challenges. Despite hurdles, including limited response rates and toxicity, ongoing clinical trials and technological advancements offer opportunities for more personalized and effective immunotherapies, which could ultimately improve outcomes for prostate cancer patients.

## 2. Penile Cancer

### 2.1. Overview of Penile Cancer

Penile cancer is a rare malignancy diagnosed typically in men over the age of 60 [49]. Although rare, it remains to be an important problem in developing nations [50]. Diagnosis can be established histologically via punch, excisional, or incisional biopsy. Squamous cell carcinoma (SCC) is the most common histological type [51]. The etiology of penile cancer is still unclear, but it is associated with human papilloma virus (HPV) subtypes 16 and 18 and a chronic inflammatory state of the foreskin and glans [50,52].

### 2.2. Current and Emerging Therapeutic Approaches

Due to poor response to first-line platinum-based chemotherapy, novel therapeutic approaches have been developed for managing penile cancer. Included in these new approaches are targeted therapy, immunotherapy, and adoptive T-cell therapy.

#### 2.2.1. Targeted Therapy

Epidermal growth factor receptor (EGFR) is highly expressed in SCC and has been targeted for therapy in penile cancer [53,54].

##### Anti-EGFR Agents

Several studies investigated Cetuximab, Panitumumab, and Nimotuzumab and have shown good results.

- Cetuximab

In a study by Vermorken et al., Cetuximab alone was given as a treatment for 103 patients who progressed after a platinum-based regimen. It showed a 13% response rate, a disease control rate of 46%, and a median survival of 178 days. Cetuximab can also enhance the outcome of platinum-based therapy, and it showed an improved response rate when used alone or in a regimen with taxane in retrospective review studies [54,55,56]. In a study by Carthon et al., cetuximab or cetuximab with platinum-based regimens showed an odds risk ratio (ORR) of 23.5%, 3.2 months median time-to-progression (TTP), and 9.8 months overall survival (OS) [55]. A case report showed cetuximab combined with anlotinib, a tyrosine kinase inhibitor (TKI), may be considered for relapsed penile SCC [57]. A few case reports also showed clinical benefits with cetuximab and chemotherapy in locally advanced penile SCC, and cetuximab 800 mg with chemotherapy and radiotherapy in one recurrent penile SCC [58,59].

- Panitumumab

Panitumumab is another anti-EGFR that has been studied for treatment of penile SCC [49]. In a study by Necchi et al., 11 patients who received at least one cisplatin-containing chemotherapy for metastatic or unresectable penile SCC were treated with Panitumumab, and they had 9.5 median OS (interquartile range [IQR] 4.9–12.6) and 1.9 months’ progression-free survival (IQR 0.9−3.0 months) [54]. A case report by Brown et al. also showed improvements in patients who were refractory to a platinum-based regimen [60].

- Nimotuzumab and Dacomitinib

The anti-EGFR monoclonal antibody Nimotuzumab also showed a partial response in a case report when combined with cisplatin-based chemotherapy [61]. Necchi et al. studied dacomitinib, a TKI that inhibits EGFR, HER1, HER2, and HER4, in the treatment of 28 chemo-naive patients with advanced penile SCC with CN2-3 or M1 disease [54]. Patients were able to tolerate treatment with 12-month OS of 54.9% and PFS of 26.2%. The 12-month OS in the locally advanced group was 64%. Complete response was seen in one patient and partial response was seen in eight patients (ORR 32.1%). To date, there is limited research on therapies targeting the EGFR signaling pathway [62].

#### 2.2.2. Immunotherapy

Programmed cell death 1 (PD-1) and programmed cell death ligand 1 (PD-L1) have also been studied in treating penile cancer. They are immune checkpoints but there is little information about PD-L1 signaling pathways in cancer cell expression [63,64]. PD-L1 is expressed by 32.1% to 51.4% of penile cancer cells and 62.4% of tumor immune infiltrating cells, and it was associated with poor survival [65,66]. In a study by Udager et al., PD-L1 expression was positively correlated with decreased cancer-specific survival and regional lymph node metastasis [67].

##### PD-1 and PD-L1 Inhibitors

Pembrolizumab is an antibody that blocks interaction of PD-1 with PD-L1 [68].

- Pembrolizumab

A case report by Chahoud et al. showed significant clinical benefit in two patients with chemotherapy refractory metastatic penile SCC [69]. The combination of pembrolizumab with stereotactic body radiation therapy in a case of recurrent metastatic penile SCC also showed a durable treatment response [70]. Three patients who progressed after treatment with paclitaxel, ifosfamide, and cisplatin were given pembrolizumab, and one of the three showed a partial response [71]. A clinical trial (NCT04357873) is currently being undertaken that uses Pembrolizumab and Vorinostat in the treatment of progressive advanced mucosal penile cancers. Pembrolizumab with the biological drug XmAb¬Æ22841 is being investigated in a clinical trial (NCT03849469) that has been recently completed.

- Durvalumab

Durvalumab is a PD-L1 inhibitor that has been used in a patient with penile SCC with locally advanced anal SCC. Durvalumab 240 mg together with chemotherapy showed a potential role of this drug in converting an unresectable disease to a curable one [72]. Durvalumab with DNA Plasmid-encoding Interleukin-12/HPV DNA Plasmids Therapeutic Vaccine INO-3112 is currently being used to check for the effectiveness of treatment in patients with stage IV penile cancer (NCT03439085).

- Atezolizumab

Atezolizumab, a PD-L1 inhibitor, was administered for two years in a patient with metastatic penile cancer who showed a near complete response after prior chemoradiation despite treatment discontinuation due to toxicity [73]. Clinical trials are ongoing to investigate PD-L1 inhibitors alone or in combination therapies, including Atezolizumab with Bevacizumab (NCT03074513), M7824-based combinations targeting TGFB1 and HPV-associated malignancies (NCT04287868), and Avelumab in platinum-resistant cases (NCT03391479). Additionally, studies are examining novel PD-L1-targeting drugs like INCB099318 (NCT04272034) and anti-PD-L1 monoclonal antibody injections (LDP) (NCT04718584) for efficacy and safety in advanced penile carcinoma.

- Nivolumab

Nivolumab is a T-cell immune checkpoint PD-1 inhibitor that has been shown to have a partial response in a patient with metastatic penile SCC [74]. Two active clinical trials (NCT03333616 and NCT02834013) are currently studying nivolumab with ipilimumab in patients with penile cancers.

- Tislelzimumab

Tislelzimumab is another humanized IgG4 anti-PD-1 monoclonal antibody that has shown potential for treating unresectable locally advanced penile SCC when combined with chemotherapy [75]. Tislelzimumab 200 mg, when combined with chemotherapy, showed promising results in a patient with postoperative recurrent penile SCC [76].

- Cemiplimab

PD-1 inhibitor Cemiplimab 350 mg was given to a patient with metastatic penile SCC. The patient previously received chemotherapy and radiotherapy and became disease-free despite the withdrawing of treatment due to SARS-CoV-2 pneumonitis.

- Sintilimab

Sintilimab is another immune checkpoint inhibitor of PD-1 with excellent treatment response in patients with penile SCC. In a study by Mei et al., two patients with advanced penile squamous cell carcinoma showed a complete response after two years of chemotherapy with sintilimab 200 mg [77]. Another case of a patient with recurrent penile cancer where a sintilimab injection was given with surgery-assisted chemotherapy also showed complete remission after four cycles of combined therapy [78]. In addition, a partial response without significant side effects was seen in a patient with metastatic poorly differentiated penile SCC who was treated with sintilimab with chemotherapy [79].

- Toripalimab

Toripalimab 240 mg, anti-PD-1, showed a partial response in a patient with recurrent metastatic penile SCC with PD-L1 expression [80].

- Erlotinib and Geftinib

The tyrosine kinase inhibitor Erlotinib was given to four patients with advanced penile SCC, and Geftinib to one patient with advanced penile SCC, in a small subset study by Carthon et al. The patients were able to tolerate treatment with promising efficacy [55].

- Sunitinib and Sorafenib

Sunitinib and Sorafenib are tyrosine kinase inhibitors that target VEGFR [81]. In a feasibility case series by Zhu et al., six patients with penile cancer received sorafenib or sunitinib after failing chemotherapy, and one had a partial response while four patients had stable disease [50]. Three of six patients showed a quality of life improvement and pain response.

- Entinostat and Bintrafusp Alfa

Entinostat, a histone deacetylase inhibitor, in combination with NHS-IL12, an immunocytokine which targets IL12, and Bintrafusp Alfa is currently being investigated in a phase I/II study (NCT04708470).

### 2.3. Combination Therapy Approaches

Combining immunotherapy has also shown promising results.

#### 2.3.1. Ipilimumab and Nivolumab

Ipilimumab and Nivolumab administered in a patient refractory to a chemotherapy regimen showed a good response [82].

#### 2.3.2. Cabozantinib and Immune Checkpoint Inhibitors

Cabozantinib, an oral TKI that inhibits anti-c-Met, VEGFR2, AXL, and RET, in combination with nivolumab and ipilimumab showed a partial response in two of three patients with advanced penile SCC [83]. Meanwhile, a Phase I trial by Nadal et al. showed two of four patients with advanced penile SCC having a partial response [84].

#### 2.3.3. Ongoing Clinical Trials

NCT02496208 is a clinical trial that is currently active which investigates a combination of Cabozantinib S-malate and Nivolumab with or without Ipilimumab in treating patients with metastatic penile cancers. Another clinical trial (NCT03866382) is also testing the effectiveness of nivolumab and ipilimumab with cabozantinib in patients with metastatic penile cancer.

### 2.4. Adoptive T-Cell Therapy in Penile Cancer

Adoptive T-cell therapy or cellular immunotherapy is an emerging penile cancer treatment that uses tumor-infiltrating lymphocytes in treating cancer [49].

#### 2.4.1. Overview of Adoptive T-Cell Therapy

This treatment expands the patient’s naturally occurring T cells ex vivo and infuses them back to target cancer cells [85]. To date, only one study published in 2021 evaluated its efficacy in a patient with metastatic SCC to the penis who received prior platinum-based therapy. The study by Doran et al. used genetically engineered T-cells against HPV16 E6, and the results support an adoptive T-cell approach in inducing regression in epithelial cancers, but continued investigation is warranted [86].

#### 2.4.2. Clinical Trials in Adoptive T-Cell Therapy

Currently, there are multiple clinical trials being performed that investigate adoptive T-cell therapy. NCT05686226 is another clinical trial investigating T-cell immunotherapy for HPV-related penile SCC. A basket study (NCT05973487) is about to begin which will investigate on T cell therapy in patients with locally advanced (unresectable) HPV-related penile cancer. Two clinical trials (NCT01585428 and NCT02280811) have been recently completed that study the effect of immunotherapy using T-cells in patients with metastatic HPV-associated penile cancer.

## 3. Testicular Cancer

### 3.1. Introduction to Testicular Cancer

Penile cancer is a rare malignancy primarily affecting men over the age of 60, with a higher prevalence in developing countries. It is often diagnosed through histological examination, with squamous cell carcinoma (SCC) as the most common subtype. Risk factors include human papillomavirus (HPV) infection and chronic inflammation of the penile foreskin and glans.

### 3.2. Immunotherapy in Testicular Cancer

#### 3.2.1. Anti-CD30 Therapy

CD30 expression has been found in testicular cancer and is associated with poor prognosis in patients with CD30-expressing embryonal carcinomas [87].

##### Brentuximab Vedotin

Brentuximab-Vedotin is an anti-CD30 antibody that is used in treatment of testicular cancers [88]. A partial response was seen in a case report which used Brentuximab-Vedotin, combined with Prembrolizumab, as a treatment in a patient with a pre-treated metastatic germ cell tumor [89]. However, the drug was discontinued after the patient developed grade 3 immune-mediated hepatitis.

##### Case Studies and Clinical Trials

In 2017, Albany et al. made a case series of seven patients with CD-30 expressing testicular cancer [87]. Specifically, five patients had germ cell tumors (GCT), one patient had a Leydig cell tumor, and one patient had a Sertoli cell tumor. They were given Brentuximab-Vedotin, which was well-tolerated and resulted in one durable complete remission and one partial response in the patients with germ cell tumors. Necchi et al. in their phase 2 trial also reported promising effects of Brentuximab-vedoitin as a salvage treatment in GCT.

#### 3.2.2. PD-1 and PD-L1 Inhibitors

In a study, Fankhauser et al. showed that PD-L1 expression can be seen in 73% of seminomas and 64% of non-seminomas, and not in normal testicular tissue. PD-1 expression was also frequent in human testicular GCT [90]. The inhibition of PD-1 and PD-L1 interaction has been investigated in multiple studies for the treatment of testicular cancer.

##### Pembrolizumab

PD-1 inhibition using Pembrolizumab has been investigated in the treatment of testicular cancer but has mixed results. A study involving 12 patients with refractory GCT showed disappointing results with pembrolizumab treatment, despite being well-tolerated [91]. Pembrolizumab was also used as a therapy in a patient with metastatic choriocarcinoma to the lung, cerebrum, and lymph nodes. However, the patient progressed rapidly with an elevation of human chorionic gonadotropin (hCG) after 200 mg pembrolizumab was given [92]. A case report was also undertaken investigating Pembrolizumab utility in a Japanese patient with chemo-refractory testicular GCT with microsatellite instability (MSI)-high. The patient had a decrease in hCG after two doses of pembrolizumab, proving the possible efficacy of the drug [93].

##### Durvalumab and Tremelimumab

Durvalumab, an anti-PD-L1, alone or in combination with tremelimumab, an anti-CTLA-4, was also studied as form of treatment in patients with advanced germ cell tumors but showed disappointing results [94].

##### Nivolumab

A case series was performed in males with refractory nonseminomatous germ cell tumors who relapsed from high-dose chemotherapy and stem cell transplantation. Seven participants were given anti-PD-1 inhibitors nivolumab or pembrolizumab. Four patients died after single-dose treatment, and one had a mixed response followed by progression. One of the seven patients achieved a partial radiographic response, while the other one had a stable disease [95]. Nivolumab was also used for 14 months in a patient with choriocarcinoma with metastasis to the lungs, bone, brain, and lymph nodes [96]. He had a partial radiographic response and B-HCG stability after treatment. In a study by Shah et al. [97] nivolumab was used in a patient with metastatic embryonal carcinoma. It showed that the Anti-PD-1 reduced tumor volume by 33%, with a decrease in serum markers supporting the use of the drug in GCT treatment.

##### Avelumab

Anti-PD-L1 Avelumab, on the other hand, was used in a phase II study in patients with multiple relapsed/refractory germ cell tumors. In the study, the drug was well-tolerated, but all patients experienced disease progression. Seven of the 15 patients died with 0% 12-week PFS, and 2.7 months median OS (95% CI 1.0–3.3) [98].

### 3.3. Targeted Therapy in Testicular Cancer

#### 3.3.1. Tyrosine Kinase Inhibitors (TKIs)

Tyrosine kinase inhibitors Sunitinib and Imatinib were studied in patients with refractory GCT but did not show a significant benefit [99,100]. Trastuzumab, an anti-HER2 drug, also did not show clinical activity against cisplatin-refractory germ-cell cancers [101].

#### 3.3.2. Anti-CTLA-4

An Ipilimumab clinical trial (NCT00060372) has been recently completed investigating persistent or progressive testicular cancer after allogeneic stem cell transplant. An Ipilimumab with Nivolumab clinical trial (NCT02834013) is currently ongoing to study its efficacy in treating rare tumors. Another clinical trial is recruiting testing the effectiveness of Ipilimumab and Nivolumab with Cabozantinib for rare tumors (NCT03866382).

### 3.4. Future Therapies and Clinical Trials

#### 3.4.1. Emerging Immunotherapies

There are multiple clinical trials on treatment of testicular cancer. A clinical trial (NCT00003408) has recently been completed investigating aldesleukin, a recombinant interleukin-2, recombinant interferon alfa, and sargramostim, a bone marrow immunostimulant as novel treatment for testicular cancer with relapse after bone marrow and peripheral stem cell transplantation. Lymphocyte infusion therapy clinical trials have also been completed in patients with testicular germ cell tumor (NCT00003887) and recurrent or high-risk non-Hodgkin lymphoma (NCT01815749) who relapsed after bone marrow and peripheral stem cell transplantation. Dendritic cell vaccine (NCT00004604) and TRICOM-CEA (6D) Vaccine (NCT00027534) are currently being investigated as therapeutic options for metastatic testicular cancers.

#### 3.4.2. Monoclonal Antibodies in Clinical Trials

Rituximab, an anti-CD20, is also being investigated on its efficacy in treating recurrent (NCT00054639), relapsed (NCT01326702), and previously treated lymphoid malignancy (NCT00072514). It was also studied in a completed clinical trial in patients undergoing donor peripheral blood stem cell transplants for relapsed or refractory B-cell lymphoma (NCT00867529). There is also a clinical trial (NCT00720135) looking at fusion protein cytokine therapy after rituximab treatment in patients with non-Hodgkin lymphoma. Alemtuzumab, an anti-CD52, was also investigated in treating patients who are undergoing donor stem cell transplantation for hematologic cancers (NCT00118352).

MORAb-004, anti-endosialin/TEM1 monoclonal antibody, has been studied in a completed clinical trial (NCT01748721) in patients with recurrent or refractory lymphoma. Lastly, Bevacizumab, an anti-VEGF, has been investigated in a recently completed clinical trial on lymphomas (NCT00458731).

## 4. Evaluating the Role and Clinical Potential of Immunotherapies and Monoclonal Antibodies in Male Reproductive Cancers

### 4.1. Immunotherapies and Monoclonal Antibodies

The clinical landscape for immunotherapies and monoclonal antibodies in male reproductive cancers is evolving rapidly, demonstrating promising results, especially in prostate cancer management. Immunotherapies like Sipuleucel-T and checkpoint inhibitors have improved outcomes in metastatic castration-resistant prostate cancer (mCRPC), although their success is tempered by moderate response rates compared to other cancer types. For rarer tumors, such as penile and testicular cancers, targeted approaches including PD-1/PD-L1 and anti-CD30 therapies have shown potential but vary in effectiveness depending on individual tumor profiles. We believe that immunotherapy’s role in these malignancies is critical as it addresses therapeutic gaps left by traditional modalities. However, treatment-related adverse effects and challenges with achieving robust responses across all tumor types highlight the need for carefully selected treatment protocols.

### 4.2. Probability of Clinical Success in Male Reproductive Tumors

The likelihood of clinical success for immunotherapies in male reproductive cancers is significant, particularly in prostate cancer, where immunotherapy has achieved regulatory approval and shown survival benefits in mCRPC cases. Success probabilities in penile and testicular cancers are somewhat lower due to the limited number of cases studied, but emerging data from checkpoint inhibitors like pembrolizumab and nivolumab in refractory cases are promising. Furthermore, adoptive T-cell therapy offers future potential in HPV-related penile cancers, with early studies supporting its efficacy.

### 4.3. Integration with Standard Therapies

The integration of immunotherapies and monoclonal antibodies within existing cancer treatment frameworks holds substantial promise. We agree that these therapies complement conventional treatments by providing additional, tumor-targeted options. Combination approaches, such as pairing checkpoint inhibitors with androgen deprivation therapy in prostate cancer, are already showing potential for improved outcomes and quality of life. In the future, incorporating immunotherapies alongside surgery, radiation, or chemotherapy in a multimodal approach could lead to more comprehensive, personalized care for patients with male reproductive cancers.

### 4.4. Conclusions

Immunotherapy and monoclonal antibodies present promising options for male reproductive cancers, particularly metastatic prostate cancer, where treatments like Sipuleucel-T and checkpoint inhibitors improve survival. While combination therapies, including CAR T-cell and BiTE, expand potential, efficacy in solid tumors remains a challenge. For rarer cancers, results vary; agents like pembrolizumab benefit select patients, emphasizing personalized treatment. Ongoing research into resistance mechanisms, biomarkers, and treatment refinement aims to enhance outcomes, advancing more effective, individualized care for male reproductive cancers.

## Figures and Tables

**Table 2:** PSMA CART cell clinical trials.

Sr. No	PSMA CART Cell Clinical Trials
1.	NCT04227275 CART-PSMA-TGFβRDN
2.	NCT01140373 Autologous anti-PSMA CAR T-cells
3.	NCT03089203 CART-PSMA-TGFβRDN
4.	NCT04249947 P-PSMA-101 CAR T-cells
5.	NCT04429451 4SCAR-PSMA T-cells
6.	NCT04633148 UniCAR02 T-cells (TMpPSMA) 39
7.	NCT04768608 non-viral PD1 integrated anti-PSMA chimeric antigen receptor T-cells
8.	NCT03692663 Anti-PSMA CAR NK cell (TABP EIC) 9
9.	NCT05354375 PSMA-targeted CAR T-cells
10.	NCT05656573 CART-PSMA cells

## References

[1] Gilliland F.D., Key C.R. (1995). Male genital cancers. Cancer.

[2] Bahlinger V., Hartmann A., Eckstein M. (2023). Immunotherapy in Genitourinary Cancers: Role of Surgical Pathologist for Detection of Immunooncologic Predictive Factors. Adv. Anat. Pathol..

[3] Yang P., Meng M., Zhou Q. (2021). Oncogenic cancer/testis antigens are a hallmarker of cancer and a sensible target for cancer immunotherapy. Biochim. Biophys. Acta Rev. Cancer.

[4] Mar N., Uchio E., Kalebasty A.R. (2022). Use of immunotherapy in clinical management of genitourinary cancers—A review. Cancer Treat. Res. Commun..

[5] Brahmer J.R., Tykodi S.S., Chow L.Q., Hwu W.J., Topalian S.L., Hwu P., Drake C.G., Camacho L.H., Kauh J., Odunsi K. (2012). Safety and activity of anti-PD-L1 antibody in patients with advanced cancer. N. Engl. J. Med..

[6] Mahoney K.M., Atkins M.B. (2014). Prognostic and predictive markers for the new immunotherapies. Oncology.

[7] Le D.T., Uram J.N., Wang H., Bartlett B.R., Kemberling H., Eyring A.D., Skora A.D., Luber B.S., Azad N.S., Laheru D. (2015). PD-1 Blockade in Tumors with Mismatch-Repair Deficiency. N. Engl. J. Med..

[8] Le D.T., Durham J.N., Smith K.N., Wang H., Bartlett B.R., Aulakh L.K., Lu S., Kemberling H., Wilt C., Luber B.S. (2017). Mismatch repair deficiency predicts response of solid tumors to PD-1 blockade. Science.

[9] Topalian S.L., Hodi F.S., Brahmer J.R., Gettinger S.N., Smith D.C., McDermott D.F., Powderly J.D., Carvajal R.D., Sosman J.A., Atkins M.B. (2012). Safety, activity, and immune correlates of anti-PD-1 antibody in cancer. N. Engl. J. Med..

[10] Kantoff P.W., Higano C.S., Shore N.D., Berger E.R., Small E.J., Penson D.F., Redfern C.H., Ferrari A.C., Dreicer R., Sims R.B. (2010). Sipuleucel-T immunotherapy for castration-resistant prostate cancer. N. Engl. J. Med..

[11] Seer Cancer Statistics. https://seer.cancer.gov/statfacts/html/prost.html.

[12] Mitsogiannis I., Tzelves L., Dellis A., Issa H., Papatsoris A., Moussa M. (2022). Prostate Cancer Immunotherapy. Expert Opin. Biol. Ther..

[13] Harsini S., Wilson D., Saprunoff H., Allan H., Gleave M., Goldenberg L., Chi K.N., Kim-Sing C., Tyldesley S., Bénard F. (2023). Outcome of Patients with Biochemical Recurrence of Prostate Cancer After PSMA PET/CT-Directed Radiotherapy or Surgery Without Systemic Therapy. Cancer Imaging.

[14] Leslie S.W., Soon-Sutton T.L., Sajjad H., Siref L.E. (2023). Prostate Cancer. StatPearls.

[15] Denmeade S.R., Isaacs J.T. (2003). Overview of Regulation of Systemic Androgen Levels. Holland-Frei Cancer Medicine.

[16] Schaeffer E.M., Srinivas S., Barocas D. NCCN Clinical Practice Guidelines in Oncology (NCCN Guidelines) Prostate Cancer. https://www.nccn.org/professionals/physician_gls/pdf/prostate.pdf.

[17] Parker C., Nilsson S., Heinrich D., Helle S.I., O’Sullivan J.M., Fosså S.D., Chodacki A., Wiechno P., Logue J., Seke M. (2013). Alpha Emitter Radium-223 and Survival in Metastatic Prostate Cancer. N. Engl. J. Med..

[18] Sartor O., de Bono J., Chi K.N., Fizazi K., Herrmann K., Rahbar K., Tagawa S.T., Nordquist L.T., Vaishampayan N., El-Haddad G. (2021). Lutetium-177–PSMA-617 for Metastatic Castration-Resistant Prostate Cancer. N. Engl. J. Med..

[19] de Bono J., Mateo J., Fizazi K., Saad F., Shore N., Sandhu S., Chi K.N., Sartor O., Agarwal N., Olmos D. (2020). Olaparib for Metastatic Castration-Resistant Prostate Cancer. N. Engl. J. Med..

[20] Pachynski R.K., Morishima C., Szmulewitz R., Harshman L., Appleman L., Monk P., Bitting R.L., Kucuk O., Millard F., Seigne J.D. (2021). IL-7 expands lymphocyte populations and enhances immune responses to sipuleucel-T in patients with metastatic castration-resistant prostate cancer (mCRPC). J. Immunother. Cancer.

[21] Small E.J., Schellhammer P.F., Higano C.S., Redfern C.H., Nemunaitis J.J., Valone F.H., Verjee S.S., Jones L.A., Hershberg R.M. (2006). Placebo-controlled phase III trial of immunologic therapy with sipuleucel-T (APC8015) in patients with metastatic, asymptomatic hormone refractory prostate cancer. J. Clin. Oncol..

[22] Higano C.S., Schellhammer P.F., Small E.J., Burch P.A., Nemunaitis J., Yuh L., Provost N., Frohlich M.W. (2009). Integrated data from 2 randomized, double-blind, placebo-controlled, phase 3 trials of active cellular immunotherapy with sipuleucel-T in advanced prostate cancer. Cancer.

[23] Anassi E., Ndefo U.A. (2011). Sipuleucel-T (Provenge) injection the first immunotherapy agent (Vaccine) for hormone-refractory prostate cancer. P & T: A peer-reviewed journal for formulary management. Pharm. Ther..

[24] Lanka S.M., Zorko N.A., Antonarakis E.S., Barata P.C. (2023). Metastatic Castration-Resistant Prostate Cancer, Immune Checkpoint Inhibitors, and Beyond. Curr. Oncol..

[25] Marabelle A., Le D.T., Ascierto P.A., Di Giacomo A.M., De Jesus-Acosta A., Delord J.-P., Geva R., Gottfried M., Penel N., Hansen A.R. (2020). Efficacy of Pembrolizumab in Patients with Noncolorectal High Microsatellite Instability/Mismatch Repair-Deficient Cancer: Results From the Phase II KEYNOTE-158 Study. J. Clin. Oncol..

[26] Sena L.A., Fountain J., Isaacsson Velho P., Lim S.J., Wang H., Nizialek E., Rathi N., Nussenzveig R., Maughan B.L., Velez M.G. (2021). Tumor Frameshift Mutation Proportion Predicts Response to Immunotherapy in Mismatch Repair-Deficient Prostate Cancer. Oncologist.

[27] Antonarakis E.S., Piulats J.M., Gross-Goupil M., Goh J., Ojamaa K., Hoimes C.J., Vaishampayan U., Berger R., Sezer A., Alanko T. (2020). Pembrolizumab for Treatment-Refractory Metastatic Castration-Resistant Prostate Cancer: Multicohort, Open-Label Phase II KEYNOTE-199 Study. J. Clin. Oncol..

[28] Kwon E.D., Drake C.G., Scher H.I., Fizazi K., Bossi A., van den Eertwegh A.J.M., Krainer M., Houede N., Santos R., Mahammedi H. (2014). Ipilimumab versus Placebo after Radiotherapy in Patients with Metastatic Castration-Resistant Prostate Cancer That Had Progressed after Docetaxel Chemotherapy (CA184-043): A Multicentre, Randomised, Double-Blind, Phase 3 Trial. Lancet Oncol..

[29] Beer T.M., Kwon E.D., Drake C.G., Fizazi K., Logothetis C., Gravis G., Ganju V., Polikoff J., Saad F., Humanski P. (2017). Randomized, Double-Blind, Phase III Trial of Ipilimumab Versus Placebo in Asymptomatic or Minimally Symptomatic Patients with Metastatic Chemotherapy-Naive Castration-Resistant Prostate Cancer. J. Clin. Oncol..

[30] Petrylak D.P., Loriot Y., Shaffer D.R., Braiteh F., Powderly J., Harshman L.C., Conkling P., Delord J.-P., Gordon M., Kim J.W. (2021). Safety and Clinical Activity of Atezolizumab in Patients with Metastatic Castration-Resistant Prostate Cancer: A Phase I Study. Clin. Cancer Res..

[31] Powles T., Yuen K.C., Gillessen S., Kadel E.E., Rathkopf D., Matsubara N., Drake C.G., Fizazi K., Piulats J.M., Wysocki P.J. (2022). Atezolizumab with enzalutamide versus enzalutamide alone in metastatic castration-resistant prostate cancer: A randomized phase 3 trial. Nat. Med..

[32] Merck News Release. https://www.merck.com/news/merck-provides-update-on-phase-3-trials-keynote-641-and-keynote-789/.

[33] Fizazi K., González Mella P., Castellano D., Minatta J.N., Rezazadeh Kalebasty A., Shaffer D., Vázquez Limón J.C., Sánchez López H.M., Armstrong A.J., Horvath L. (2022). Nivolumab Plus Docetaxel in Patients with Chemotherapy-Naïve Metastatic Castration-Resistant Prostate Cancer: Results from the Phase II CheckMate 9KD Trial. Eur. J. Cancer.

[34] Yu E.Y., Kolinsky M.P., Berry W.R., Retz M., Mourey L., Piulats J.M., Appleman L.J., Romano E., Gravis G., Gurney H. (2022). Pembrolizumab Plus Docetaxel and Prednisone in Patients with Metastatic Castration-Resistant Prostate Cancer: Long-Term Results from the Phase 1b/2 KEYNOTE-365 Cohort B Study. Eur. Urol..

[35] Petrylak D.P., Li B., Schloss C., Fizazi K. (2019). KEYNOTE-921: Phase III Study of Pembrolizumab (Pembro) Plus Docetaxel and Prednisone for Enzalutamide (Enza)- or Abiraterone (Abi)-Pretreated Patients (Pts) with Metastatic Castration-Resistant Prostate Cancer (MCRPC). Ann. Oncol..

[36] Sharma P., Pachynski R.K., Narayan V., Fléchon A., Gravis G., Galsky M.D., Mahammedi H., Patnaik A., Subudhi S.K., Ciprotti M. (2020). Nivolumab Plus Ipilimumab for Metastatic Castration-Resistant Prostate Cancer: Preliminary Analysis of Patients in the CheckMate 650 Trial. Cancer Cell.

[37] Sharma P., Krainer M., Saad F., Castellano D., Bedke J., Kwiatkowski M., Patnaik A., Procopio G., Wiechno P., Kochuparambil S.T. (2023). Nivolumab plus Ipilimumab for the Treatment of Post-Chemotherapy Metastatic Castration-Resistant Prostate Cancer (MCRPC): Additional Results from the Randomized Phase 2 CheckMate 650 Trial. J. Clin. Oncol..

[38] He M., Zhang D., Cao Y., Chi C., Zeng Z., Yang X., Yang G., Sharma K., Hu K., Enikeev M. (2023). Chimeric antigen receptor-modified T cells therapy in prostate cancer: A comprehensive review on the current state and prospects. Heliyon.

[39] Schepisi G., Cursano M.C., Casadei C., Menna C., Altavilla A., Lolli C., Cerchione C., Paganelli G., Santini D., Tonini G. (2019). CAR-T cell therapy: A potential new strategy against prostate cancer. J. Immunother. Cancer.

[40] Zuccolotto G., Fracasso G., Merlo A., Montagner I.M., Rondina M., Bobisse S., Figini M., Cingarlini S., Colombatti M., Zanovello P. (2014). PSMA-specific CAR-engineered T cells eradicate disseminated prostate cancer in preclinical models. PLoS ONE.

[41] Wang D., Shao Y., Zhang X., Lu G., Liu B. (2020). IL-23 and PSMA-targeted duo-CAR T cells in Prostate Cancer Eradication in a preclinical model. J. Transl. Med..

[42] Narayan V., Barber-Rotenberg J.S., Jung I.-Y., Lacey S.F., Rech A.J., Davis M.M., Hwang W.-T., Lal P., Carpenter E.L., Maude S.L. (2022). PSMA-targeting TGFβ-insensitive armored CAR T cells in metastatic castration-resistant prostate cancer: A phase 1 trial. Nat. Med..

[43] Rosa K. P-PSMA-101 Elicits Encouraging Responses in Metastatic Castration-Resistant Prostate Cancer. https://www.onclive.com/view/p-psma-101-elicits-encouraging-responses-in-metastatic-castration-resistant-prostate-cancer.

[44] Priceman S.J., Gerdts E.A., Tilakawardane D., Kennewick K.T., Murad J.P., Park A.K., Jeang B., Yamaguchi Y., Yang X., Urak R. (2018). Co-stimulatory signaling determines tumor antigen sensitivity and persistence of CAR T cells targeting PSCA+ metastatic prostate cancer. OncoImmunology.

[45] Moradi A., Srinivasan S., Clements J., Batra J. (2019). Beyond the biomarker role: Prostate-specific antigen (PSA) in the prostate cancer microenvironment. Cancer Metastasis Rev..

[46] Park H., Lee S.W., Song G., Kang T.W., Jung J.H., Chung H.C., Kim S.J., Park C.-H., Park J.Y., Shin T.Y. (2018). Diagnostic performance of %[-2]proPSA and prostate Health Index for prostate cancer: Prospective, multi-institutional study. J. Korean Med. Sci..

[47] Zarrabi K.K., Narayan V., Mille P.J., Zibelman M.R., Miron B., Bashir B., Kelly W.K. (2023). Bispecific PSMA antibodies and CAR-T in metastatic castration-resistant prostate cancer. Ther. Adv. Urol..

[48] Slovin S.F., Dorff T.B., Falchook G.S., Wei X.X., Gao X., McKay R.R., Oh D.Y., Wibmer A.G., Spear M.A., McCaigue J. (2022). Phase 1 study of P-PSMA-101 CAR-T cells in patients with metastatic castration-resistant prostate cancer (mCRPC). J. Clin. Oncol..

[49] White J., Mason R., Lawen T., Spooner J., Faria K.V., Rahman F., Ramasamy R. (2023). Therapeutic approaches to penile cancer: Standards of care and recent developments. Research and Reports in Urology. Res. Rep. Urol..

[50] Zhu Y., Li H., Yao X.-D., Zhang S.-L., Zhang H.-L., Shi G.-H., Yang L.-F., Yang Z.-Y., Wang C.-F., Ye D.-W. (2010). Feasibility and activity of sorafenib and sunitinib in advanced penile cancer: A preliminary report. Urol. Int..

[51] Giona S., Barber N., Ali A. (2022). The epidemiology of penile cancer. Urologic Cancers.

[52] Minhas S., Manseck A., Watya S., Hegarty P.K. (2010). Penile cancer–prevention and premalignant conditions. Urology.

[53] Chaux A., Munari E., Katz B., Sharma R., Lecksell K., Cubilla A.L., Burnett A.L., Netto G.J. (2013). The Epidermal Growth Factor Receptor Is Frequently Overexpressed in Penile Squamous Cell Carcinomas: A Tissue Microarray and Digital Image Analysis Study of 112 Cases. Hum. Pathol..

[54] Necchi A., Giannatempo P., Vullo S.L., Raggi D., Nicolai N., Colecchia M., Perrone F., Mariani L., Salvioni R. (2016). Panitumumab treatment for advanced penile squamous cell carcinoma when surgery and chemotherapy have failed. Clin. Genitourin. Cancer.

[55] Carthon B.C., Ng C.S., Pettaway C.A., Pagliaro L.C. (2014). Epidermal Growth Factor Receptor-Targeted Therapy in Locally Advanced or Metastatic Squamous Cell Carcinoma of the Penis. BJU Int..

[56] Vermorken J.B., Trigo J., Hitt R., Koralewski P., Diaz-Rubio E., Rolland F., Knecht R., Amellal N., Schueler A., Baselga J. (2007). Open-label, uncon-trolled, multicenter phase II study to evaluate the efficacy and toxicity of cetuximab as a single agent in patients with recurrent and/or metastatic squamous cell carcinoma of the head and neck who failed to respond to platinum-based therapy. J. Clin. Oncol..

[57] Dai S., Liu Y., Liu T., Zhang Y., Luo D. (2022). Case report of penile cancer recurrence treated with cetuximab combined with anlotinib. Clin. Case Rep..

[58] Liu J.-Y., Luo W.-X., He J.-P., Li X. (2015). Neoadjuvant chemotherapy with cetuximab for locally advanced penile cancer. J. Cancer Res. Ther..

[59] Wu J., Cheng K., Yuan L., Du Y., Li C., Chen Y., Yu Y., Gou H., Xu F., Liu J. (2016). Recurrent Penile Squamous Cell Carcinoma Successfully Treated with Cetuximab, Chemotherapy, and Radiotherapy. Clin. Genitourin. Cancer.

[60] Brown A., Ma Y., Danenberg K., Schuckman A.K., Pinski J.K., Pagliaro L.C., Quinn D.I., Dorff T.B. (2014). Epidermal growth factor receptor-targeted therapy in squamous cell carcinoma of the penis: A report of 3 cases. Urology.

[61] Men H.-T., Gou H.-F., Qiu M., He J.-P., Cheng K., Chen Y., Ge J., Liu J.-Y. (2014). A case of penile squamous cell carcinoma treated with a combination of antiepidermal growth factor receptor antibody and chemotherapy. Anticancer. Drugs.

[62] Peyraud F., Allenet C., Gross-Goupil M., Domblides C., Lefort F., Daste A., Yacoub M., Haaser T., Ferretti L., Robert G. (2020). Current management and future perspectives of penile cancer: An updated review. Cancer Treat. Rev..

[63] Junker K., Eckstein M., Fiorentino M., Montironi R. (2020). PD1/PD-L1 axis in uro-oncology. Curr. Drug Targets.

[64] Seliger B. (2019). Basis of PD1/PD-L1 therapies. J. Clin. Med..

[65] Davidsson S., Carlsson J., Giunchi F., Harlow A., Kirrander P., Rider J., Fiorentino M., Andrén O. (2019). PD-L1 Expression in Men with Penile Cancer and Its Association with Clinical Outcomes. Eur. Urol. Oncol..

[66] De Bacco M.W., Carvalhal G.F., MacGregor B., Marçal J.M., Wagner M.B., Sonpavde G.P., Fay A.P. (2020). PD-L1 and P16 Expression in Penile Squamous Cell Carcinoma From an Endemic Region. Clin. Genitourin. Cancer.

[67] Udager A.M., Liu T.-Y., Skala S.L., Magers M.J., McDaniel A.S., Spratt D.E., Feng F.Y., Siddiqui J., Cao X., Fields K.L. (2016). Frequent PD-L1 expression in primary and metastatic penile squamous cell carcinoma: Potential opportunities for immunotherapeutic approaches. Ann. Oncol..

[68] Fessas P., Lee H., Ikemizu S., Janowitz T. (2017). A molecular and preclinical comparison of the PD-1–targeted T-cell check-point inhibitors nivolumab and pembrolizumab. Semin. Oncol..

[69] Chahoud J., Skelton W.P., Spiess P.E., Walko C., Dhillon J., Gage K.L., Johnstone P.A.S., Jain R.K. (2020). Case report: Two cases of chemotherapy refractory metastatic penile squamous cell carcinoma with extreme durable response to pembrolizumab. Front. Oncol..

[70] Kaakour D., Seyedin S., Houshyar R., Mar N. (2022). Combination of Pembrolizumab and Stereotactic Body Radiation Therapy in Recurrent Metastatic Penile Squamous Cell Carcinoma: A Case Study. Biomedicines.

[71] Hahn A.W., Chahoud J., Campbell M.T., Karp D.D., Wang J., Stephen B., Tu S.-M., Pettaway C.A., Naing A. (2021). Pembrolizumab for advanced penile cancer: A case series from a phase II basket trial. Investig. New Drugs.

[72] Chen H.X., Lin C., Lin C.C., Yang C. (2022). Combination of durvalumab and chemotherapy to potentially convert unresectable stage IV penile squamous cell carcinoma to resectable disease: A case report. Curr. Oncol..

[73] Hui G., Ghafouri S.N., Shen J., Liu S., Drakaki A. (2019). Treating penile cancer in the immunotherapy and targeted therapy era. Case Rep. Oncol. Med..

[74] Trafalis D.T., Alifieris C.E., Kalantzis A., Verigos K.E., Vergadis C., Sauvage S. (1997). Evidence for efficacy of treatment with the anti-PD-1 mab nivolumab in radiation and multichemorefractory advanced penile squamous cell carcinoma. J. Immunother..

[75] Long X.Y., Zhang S., Tang L.S., Li X., Liu J.Y. (2022). Conversion therapy for advanced penile cancer with tislelizumab combined with chemotherapy: A case report and review of literature. World J. Clin. Cases.

[76] Li N., Xu T., Zhou Z., Li P., Jia G., Li X. (2022). Immunotherapy Combined with Chemotherapy for Postoperative Recurrent Penile Squamous Cell Carcinoma: A Case Report and Literature Review. Front. Oncol..

[77] Mei X., Zhao Y., Zhang Y., Liao J., Jiang C., Qian H., Du Y. (2022). Efficacy and Biomarker Exploration of Sintilimab Combined with Chemotherapy in the Treatment of Advanced Penile Squamous Cell Carcinoma-A Report of Two Cases. Front. Oncol..

[78] Hu L., Shan X., Han D., Guo Z., Wang H., Xiao Z. (2021). Multimodal Treatment Combining Salvage Surgery-Assisted Chemotherapy and Checkpoints Blockade Immunotherapy Achieves Complete Remission on a Recurrent Penile Cancer Patient: A Case Report. OncoTargets Ther..

[79] Du Y., Zhang X., Zhang Y., Li W., Hu W., Zong L., Zhao J. (2022). PD-1 inhibitor treatment in a penile cancer patient with MMR/MSI status heterogeneity: A case report. Hum. Vaccines Immunother..

[80] Su X., Zhang J., Fu C., Xiao M., Wang C. (2020). Recurrent Metastatic Penile Cancer Patient with Positive PD-L1 Expression Obtained Significant Benefit from Immunotherapy: A Case Report and Literature Review. OncoTargets Ther..

[81] Wilhelm S.M., Carter C., Tang L., Wilkie D., McNabola A., Rong H., Chen C., Zhang X., Vincent P., McHugh M. (2004). BAY 43-9006 exhibits broad spectrum oral antitumor activity and targets the RAF/MEK/ERK pathway and receptor tyrosine kinases involved in tumor progression and angiogenesis. Cancer Res..

[82] Baweja A., Mar N. (2021). Metastatic penile squamous cell carcinoma with dramatic response to combined checkpoint blockade with ipilimumab and nivolumab. J. Oncol. Pharm. Pract..

[83] Apolo A.B., Nadal R., Girardi D.M., Niglio S.A., Ley L., Cordes L.M., Steinberg S.M., Ortiz O.S., Cadena J., Diaz C. (2020). Phase I study of cabozantinib and nivolumab alone or with ipilimumab for advanced or metastatic urothelial carcinoma and other genitourinary tumors. J. Clin. Oncol..

[84] Nadal R.M., Mortazavi A., Stein M., Pal S.K., Davarpanah N.N., Parnes H.L., Ning Y.M., Cordes L.M., Bagheri M.H., Lindenberg L. (2018). Results of phase I plus expansion cohorts of cabozantinib (Cabo) plus nivolumab (Nivo) and CaboNivo plus ipilimumab (Ipi) in patients (pts) with with metastatic urothelial carcinoma (mUC) and other genitourinary (GU) malignancies. J. Clin. Oncol..

[85] Perica K., Varela J.C., Oelke M., Schneck J. (2015). Adoptive T cell immunotherapy for cancer. Rambam Maimonides Med. J..

[86] Doran S.L., Stevanović S., Adhikary S., Gartner J.J., Jia L., Kwong M.L.M., Faquin W.C., Hewitt S.M., Sherry R.M., Yang J.C. (2019). T-Cell Receptor Gene Therapy for Human Papillomavirus-Associated Epithelial Cancers: A First-In-Human, Phase I/II Study. J. Clin. Oncol..

[87] Albany C., Einhorn L., Garbo L., Boyd T., Josephson N., Feldman D.R. (2018). Treatment of CD30-Expressing Germ Cell Tumors and Sex Cord Stromal Tumors with Brentuximab Vedotin: Identification and Report of Seven Cases. Oncologist.

[88] Necchi A., Magazzu D., Anichini A., Raggi D., Giannatempo P., Nicolai N., Colecchia M., Paolini B., Coradeschi E., Tassi E. (2016). An open-label, single-group, phase 2 study of brentuximab vedotin as salvage therapy for males with relapsed germ cell tumors (GCT): Results at the end of first stage (FM12GCT01). J. Clin. Oncol..

[89] Mayrhofer K., Strasser-Weippl KNiedersüß-Beke D. (2018). Pembrolizumab plus brentuximab-vedotin in a patient with pretreated metastatic germ cell tumor. Ann. Hematol. Oncol..

[90] Fankhauser C.D., Curioni-Fontecedro A., Allmann V., Beyer J., Tischler V., Sulser T., Moch H., Bode P.K. (2015). Frequent PD-L1 expression in testicular germ cell tumors. Br. J. Cancer.

[91] Adra N., Einhorn L., Althouse S., Ammakkanavar N., Musapatika D., Albany C., Vaughn D., Hanna N. (2018). Phase II trial of pembrolizumab in patients with platinum refractory germ-cell tumors: A Hoosier Cancer Research Network Study GU14-206. Ann. Oncol..

[92] Loh K.P., Fung C. (2017). Novel therapies in platinum-refractory metastatic germ cell tumor: A case report with a focus on a PD-1 inhibitor. Rare Tumors.

[93] Kawai K., Tawada A., Onozawa M., Inoue T., Sakurai H., Mori I., Takiguchi Y., Miyazaki J. (2021). Rapid Response to Pembrolizumab in a Chemo-Refractory Testicular Germ Cell Cancer with Microsatellite Instability-High. OncoTargets Ther..

[94] Necchi A., Giannatempo P., Raggi D., Mariani L., Colecchia M., Farè E., Monopoli F., Calareso G., Ali S.M., Ross J.S. (2019). An open-label randomized phase 2 study of Durvalumab alone or in combination with tremelimumab in patients with advanced germ cell tumors (APACHE): Results from the first planned interim analysis. Eur. Urol..

[95] Zschäbitz S., Lasitschka F., Hadaschik B., Hofheinz R.-D., Jentsch-Ullrich K., Grüner M., Jäger D., Grüllich C. (2017). Response to anti-programmed cell death protein-1 antibodies in men treated for platinum refractory germ cell cancer relapsed after high- dose chemotherapy and stem cell transplantation. Eur. J. Cancer.

[96] Chi E.A., Schweizer M.T. (2017). Durable response to immune checkpoint blockade in a platinum-refractory patient with nonseminomatous germ cell tumor. Clin. Genitourin. Cancer.

[97] Shah S., Ward J.E., Bao R., Hall C.R., Brockstein B.E., Luke J.J. (2016). Clinical response of a patient to Anti-PD-1 immunotherapy and the immune landscape of testicular germ cell tumors. Cancer Immunol. Res..

[98] Mego M., Svetlovska D., Chovanec M., Rečkova M., Rejlekova K., Obertova J., Palacka P., Sycova-Mila Z., De Giorgi U., Mardiak J. (2019). Phase II study of avelumab in multiple relapsed/refractory germ cell cancer. Investig. New Drugs.

[99] Feldman D.R., Turkula S., Ginsberg M.S., Ishill N., Patil S., Carousso M., Bosl G.J., Motzer R.J. (2010). Phase II trial of sunitinib in patients with relapsed or refractory germ cell tumors. Investig. New Drugs.

[100] Einhorn L.H., Brames M.J., Heinrich M.C., Corless C.L., Madani A. (2006). Phase II study of imatinib mesylate in chemotherapy refractory germ cell tumors expressing KIT. Am. J. Clin. Oncol..

[101] Kollmannsberger C., Pressler H., Mayer F., Kanz L., Bokemeyer C. (1999). Cisplatin-refractory, HER2/neu-expressing germ-cell cancer: Induction of remission by the monoclonal antibody Trastuzumab. Ann. Oncol..

